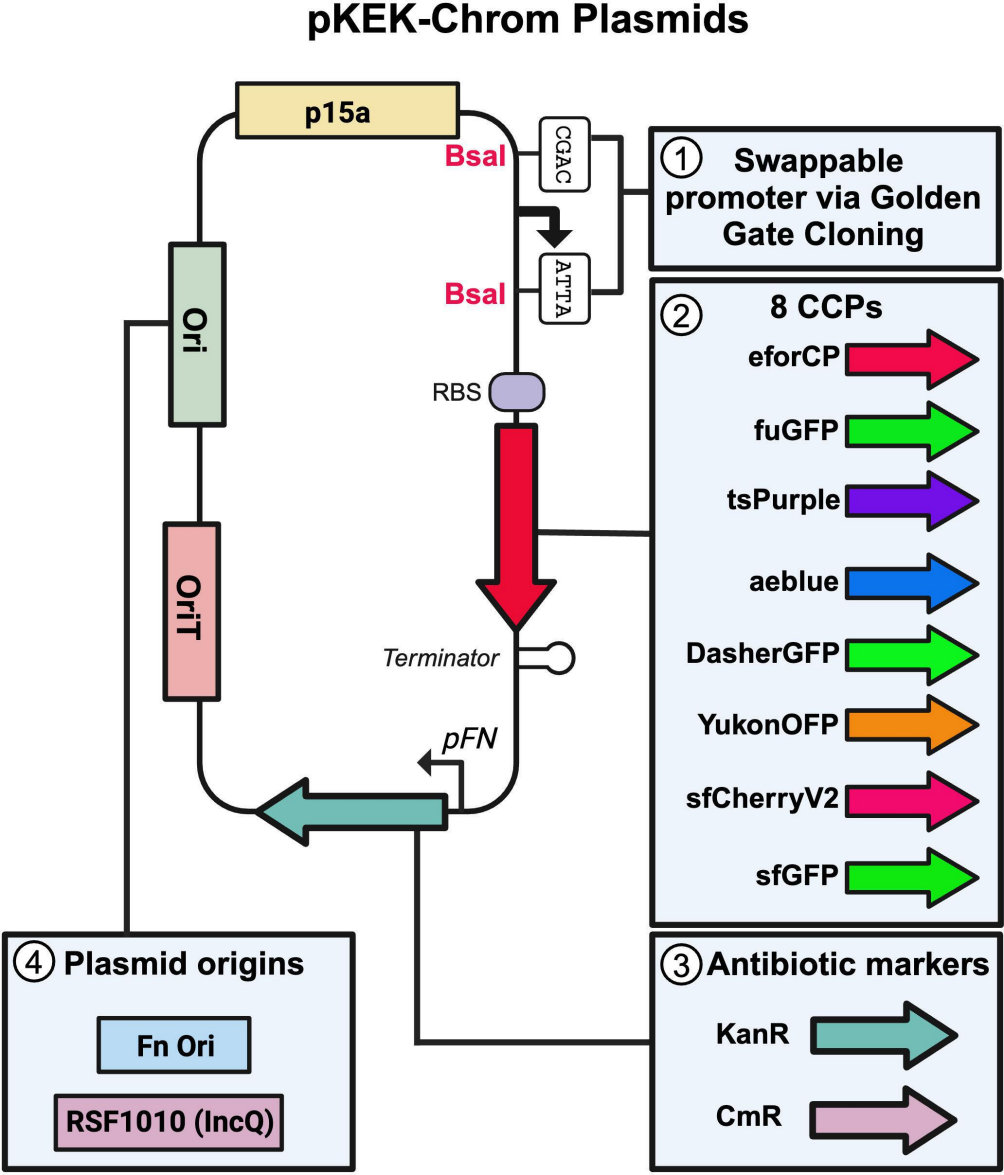# Articles of Significant Interest in This Issue

**DOI:** 10.1128/aem.01148-25

**Published:** 2025-06-18

## THE COMING OF AGE OF THE HUMAN PHAGEOME

Perturbations of the human phageome serve as early indicators of gut dysbiosis and other disorders. Rybicka and Kaźmierczak (e01788-24) review the intricate interactions between phages, bacteria, and the human host which can be harnessed as diagnostic or therapeutic tools.


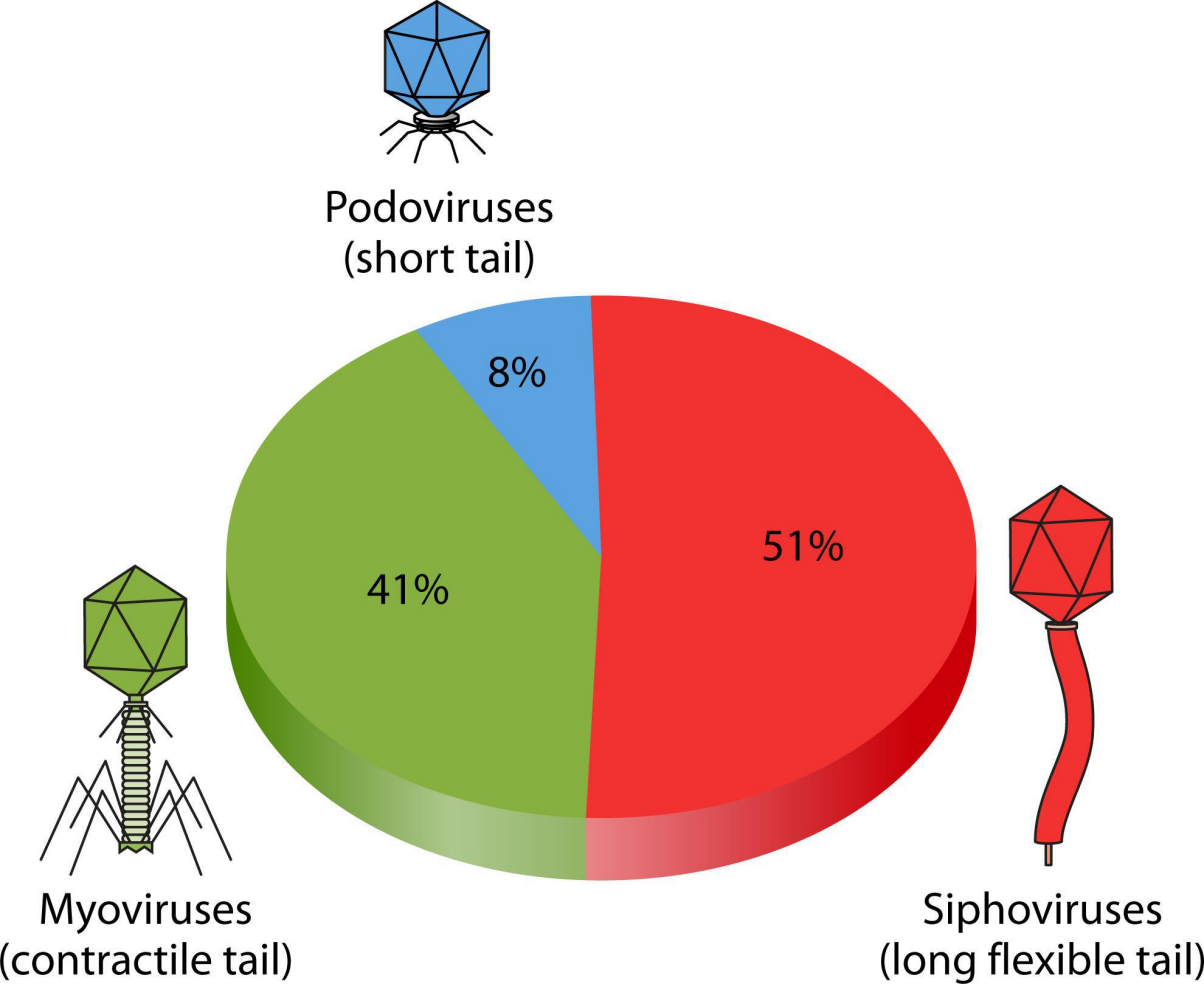


## A GENOMIC VIEW OF SYMBIOSIS IN SUCKING LICE

Říhová et al. (e00220-25) sequenced the metagenome of the understudied *Rhynchophthirina* lice to identify a symbiont with a severely reduced genome yet retention of functional pathways for the synthesis of vitamins needed by blood-feeding insects.


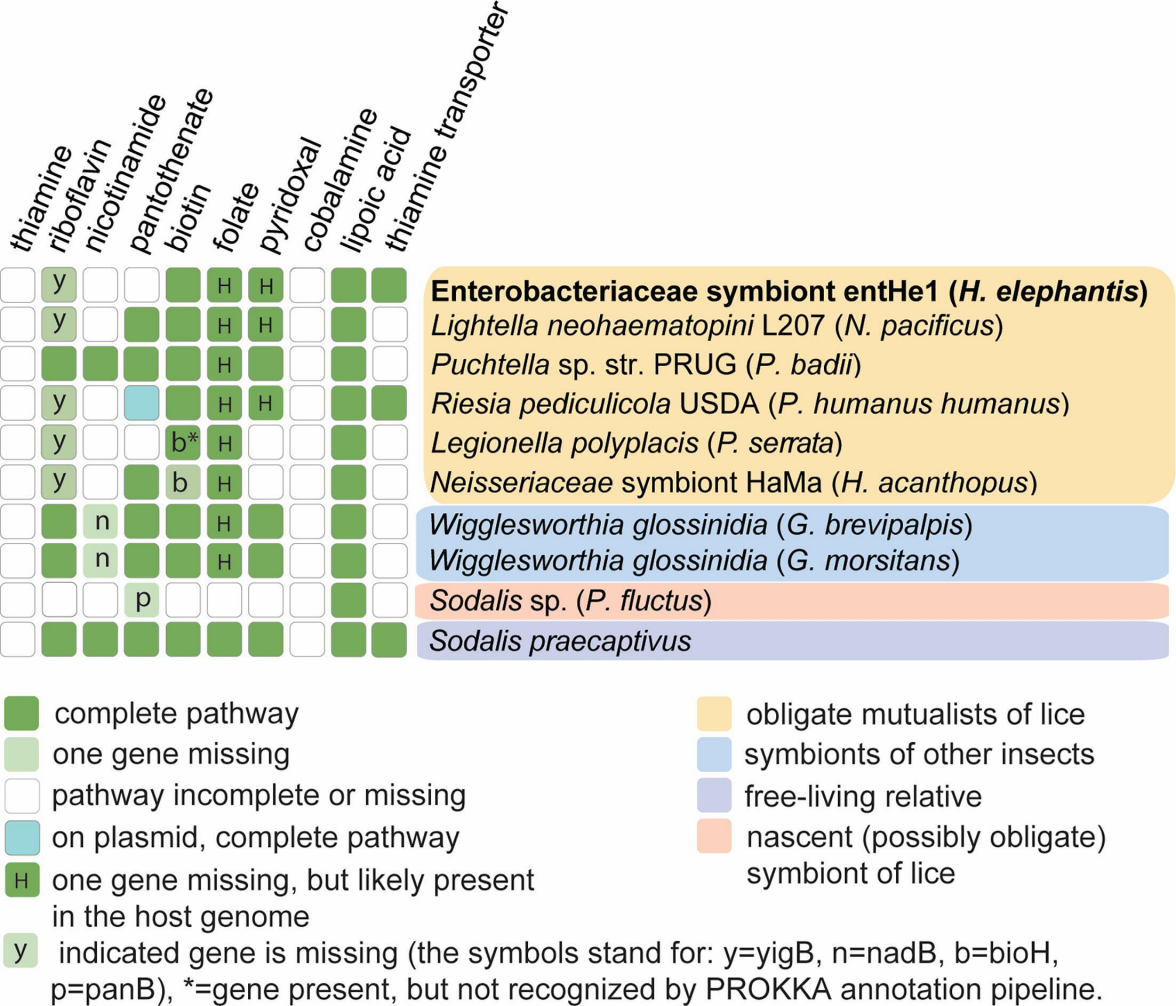


## TO BUFFER OR NOT TO BUFFER

This minireview by Prakash et al. (e01728-24) warns about detrimental impacts of buffering systems on microbial cultivation and discusses the benefits of unbuffered media formulations in applied and environmental research.


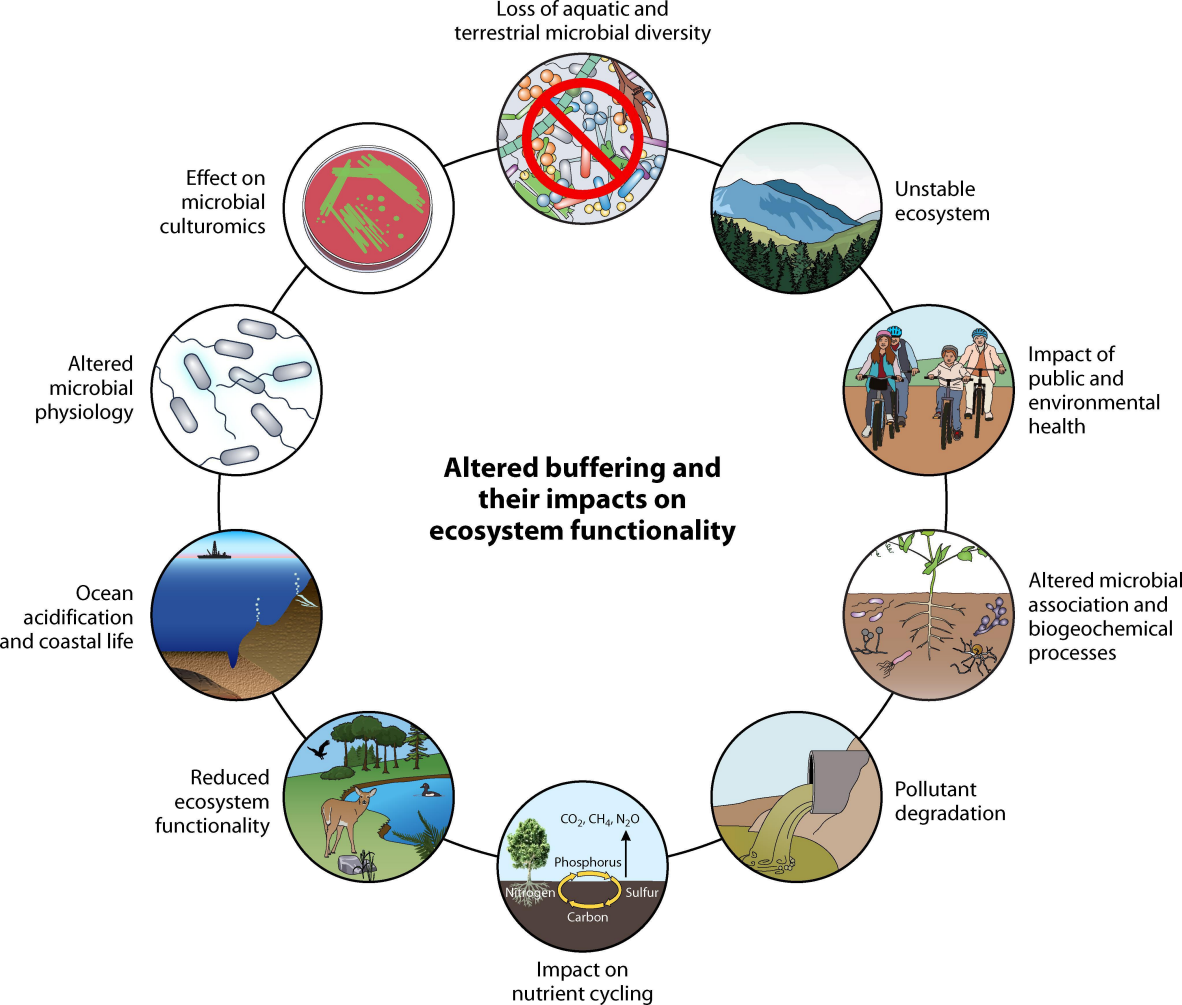


## PERSISTENCE OF ANTIBIOTIC RESISTANCE IN DRUG-FREE LIVESTOCK FARMS

Pfeifer et al. (e01386-24) show that certain resistant strains of *Escherichia coli* can persist in pig farms long after restricting antibiotic usage. Targeted action is needed against those specific strains.


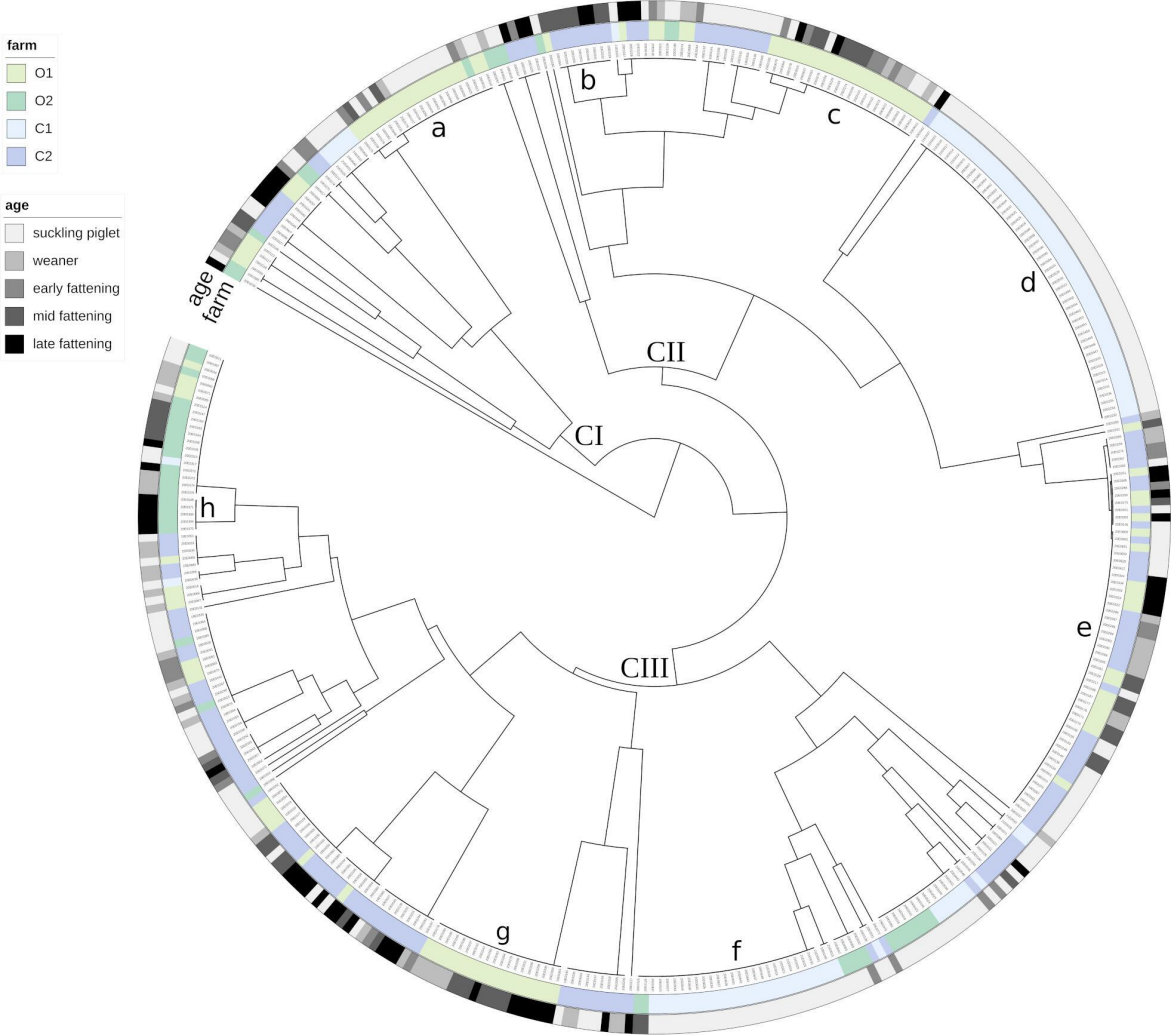


## SynCom GOES ANAEROBIC

Synthetic microbial communities (SynComs) facilitate the study of complex microbiomes. This article by Jourdain and Gu (e00404-25) in the Anaerobic Microbiology Special Series reviews the SynCom design principles and their utility to enhance anaerobic waste treatment.


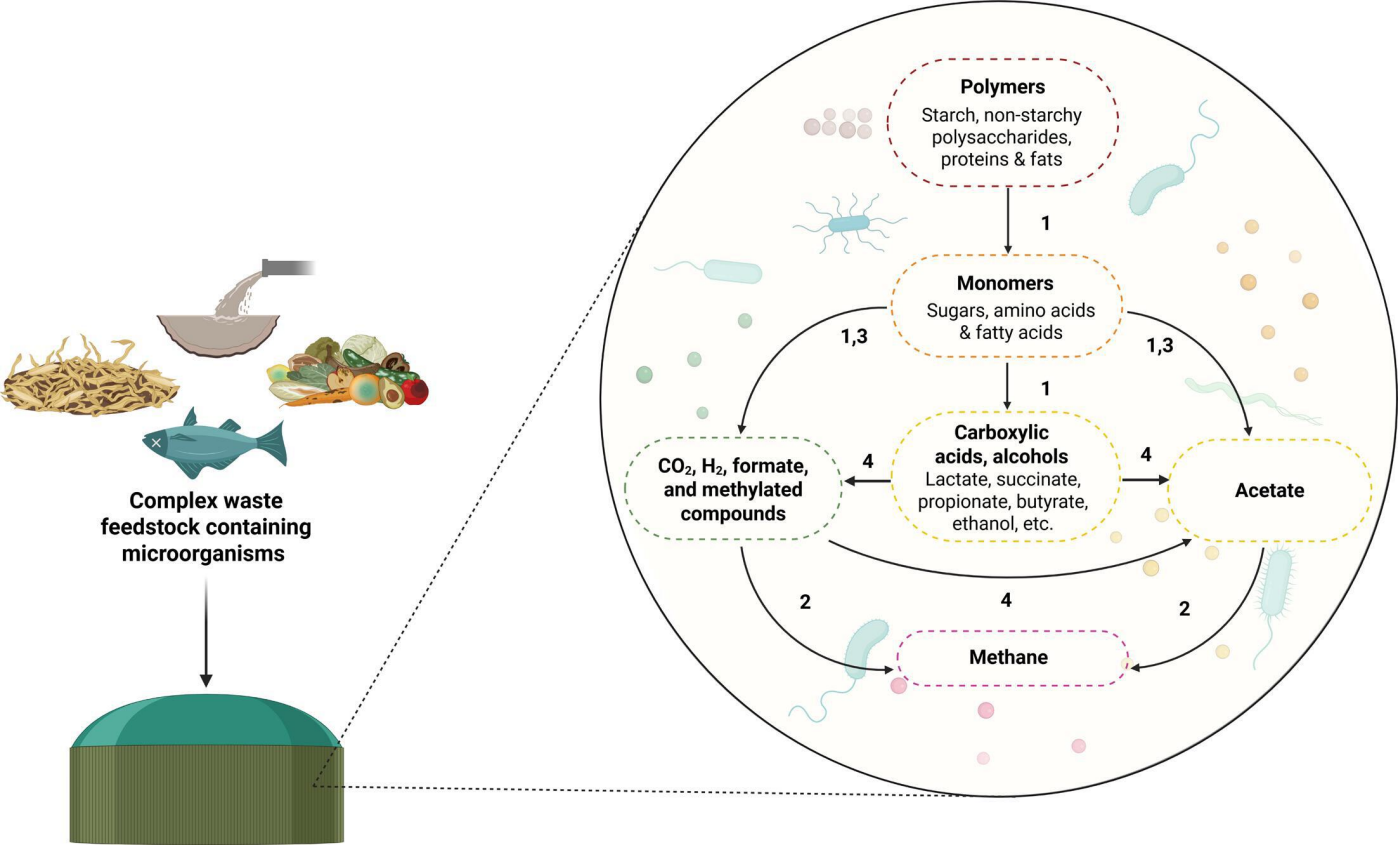


## SHUTTLE VECTORS LIGHTING THE WAY

Armstrong et al. (e00045-25) constructed a large inventory of chromophore-expressing shuttle vectors to advance genetic studies in several non-model, Gram-negative bacteria.